# Metagenomes of Maize Rhizosphere Samples after Different Fertilization Treatments at Molelwane Farm, Located in North-West Province, South Africa

**DOI:** 10.1128/MRA.00937-20

**Published:** 2020-10-22

**Authors:** Olubukola Oluranti Babalola, Matthew Chekwube Enebe

**Affiliations:** a Food Security and Safety Niche Area, Faculty of Natural and Agricultural Sciences, North-West University, Mmabatho, South Africa; Indiana University, Bloomington

## Abstract

The need for sustainable agriculture is a global issue that requires urgent attention, particularly in the areas of soil fertility enhancement and management. In this study, the effects of organic and inorganic fertilizers on the rhizosphere microbial communities of maize plants were evaluated.

## ANNOUNCEMENT

Increases in crop yields through soil fertilization ensure the sustainability of human food and livestock feed supplies. Over the years, chemical fertilizers have been used to increase soil fertility for maximum crop yield, but they have drawbacks, ranging from soil salinization, acidification, and reduction of soil quality to eutrophication (1, 2). This raises interest regarding the adoption of alternative and more sustainable means of soil nutrient enrichment through the use of organic fertilizer or composted manure (3, 4).

Rhizosphere soil samples from maize plants used in this study were sourced from an experimental field of North-West University located at the Molelwane Farm in North-West Province, South Africa (25°47′24.17604″S, 25°37′9.08328″E; 25°47′29.97048″S, 25°37′8.62428″E; 25°47′23.9604″S, 25°37′8.43348″E; 25°47′23.82252′S, 25°37′8.30064″E; and 25°47′24.11844″S, 25°37′8.18148″E; altitude, 1,012 m), with a temperature range of 22°C to 35°C and an average annual rainfall of 450 mm (5). The stabilization period for the compost prior to its use was 16 weeks. The soil was fertilized in early spring (September), followed by planting of the maize seeds (midaltitude variety of maize, *Zea mays everta*). Rhizosphere soil samples were collected at depths of 0 to 15 cm using an auger, 2 cm from the growing maize plants, at 7 weeks after germination. Sampling distances of 15 cm (minimum) to 50 cm (maximum) in 3 sampling points/plot were used. The sampling area was split into 3 plots for 60 kg N/ha and 3 plots for 120 kg N/ha. The control and compost treatments were split into 3 plots each. Soil samples were taken from 9 plants (3 from each replicate/treatment). Therefore, a total of 15 samples were collected (3 replicates × 5 treatments). From each subreplicated plot for the treatments, 9 subsamples that were distributed to cover the entire plot were gathered together as composite samples. Each of the 5 treatments was made up of 5 composite samples, each containing 9 subsamples. The collected samples were put in sterile plastic bags inside a box containing ice and were transported to the laboratory. Plant and root debris was sieved out using a sieve with a 2-mm pore size, and the samples were stored at −20°C for metagenomic shotgun analysis.

The microbial community DNA was extracted from soil using the PowerSoil DNA isolation kit from Mo Bio Laboratories, Inc., according to the manufacturer’s protocol. The extracted DNA was processed through a shotgun metagenomics sequencing procedure at MR DNA (Shallowater, TX, USA). The quantity of DNA for the analysis was evaluated using the Qubit double-stranded DNA (dsDNA) high-sensitivity (HS) assay kit (Life Technologies, Carlsbad, CA, USA). Then, following the manufacturer’s user guide, DNA libraries were prepared using the Nextera DNA Flex library preparation kit (Illumina). A total of 50 ng DNA from each sample was used to prepare the libraries. The samples were passed through the fragmentation processes in the presence of an added adapter sequence. Adapters were used during the 6 cycles of a limited PCR procedure in which unique indices were introduced into the sample. To measure the final library concentrations obtained, the Qubit dsDNA HS assay kit was used; this was followed by determination of the average library size with the use of a 2100 Bioanalyzer (Agilent Technologies, Santa Clara, CA, USA). DNA libraries were then combined in an equimolar ratio at 0.7 nM, followed by 300 cycles of paired-end sequencing using the NovaSeq 6000 system (Illumina).

The raw metagenomic reads were uploaded to MG-RAST, where quality control procedures were performed on the reads (6). Preprocessing of the reads involved removal of artificial reads and other ambiguous base pairs; this was followed by gene annotation using the BLAT algorithm (7) and the M5NR database (8). Protein-coding gene annotation was executed by searching the M5NR database using BLAST, as well as using the SEED subsystem-level function. The microbial taxa were determined by comparing the sequences against the RefSeq database using BLAST. The BLASTX tool was used to search protein databases using a translated nucleotide query with an E value cutoff of 10E−5, a minimum alignment length of 15 bp, and a percent identity of 60%. The MG-RAST normalization tool was applied in order to make more meaningful comparisons and to enable visual exploration of data such as beta diversity, as well as for clustering analyses. The sequence data generated before and after quality control are presented in Table 1. These data were analyzed statistically using Canoco version 5 and PAST version 3 (9). Default parameters were used except where otherwise noted. The reads contain 98.2% bacteria, 1.2% eukaryotes, 0.2% viruses, and 0.4% archaea. The predominant bacterial phyla were *Proteobacteria* and *Actinobacteria.* Eukaryotes and viruses were most abundant in composted-manure-treated soil. Functional analysis of the metagenomes showed that they contained genes involved in carbon, nitrogen, and phosphorus cycling, as well as antimicrobial substance-producing genes that participate in disease-suppressing soil development.

**TABLE 1:** Sequence data for the rhizosphere soil samples analyzed after treatments

Sample^*a*^	Data before QC^*b*^					Data after QC				Data after processing		Data after alignment	
	Size (bp)	No. of sequences	Sequence length (mean ± SD) (bp)	GC content (mean ± SD) (%)	No. of artificial duplicate reads	Size (bp)	No. of sequences	Sequence length (mean ± SD) (bp)	GC content(mean ± SD) (%)	No. of protein features predicted	No. of rRNA features predicted	No. of protein features identified	No. of rRNA features identified
Cp4	1,939,891,250	12,070,719	161 ± 73	49 ± 11	8,391,961	526,025,382	2,945,816	179 ± 71	44 ± 10	1,342,578	18,398	387,527	2,577
Cp8	2,618,758,280	15,575,330	168 ± 71	63 ± 12	1,832,282	2,270,368,498	13,083,355	174 ± 67	63 ± 10	11,042,146	36,758	4,547,525	10,755
N2	978,960,789	5,558,478	176 ± 74	43 ± 11	2,399,030	553,971,710	2,892,203	192 ± 71	41 ± 9	1,164,182	13,662	418,097	3,170
N1	1,694,792,733	9,687,815	175 ± 72	64 ± 12	1,078,716	1,474,813,072	8,198,530	180 ± 68	65 ± 10	7,188,585	18,817	3,057,707	6,303
Cn0	1,430,407,056	7,834,687	183 ± 70	64 ± 11	788,921	1,257,716,569	6,780,803	185 ± 67	64 ± 10	6,123,837	17,813	2,612,059	5,936

a Cp4, 4 tons/ha compost; Cp8, 8 tons/ha compost; N1, 60 kg/ha inorganic fertilizer; N2, 120 kg/ha inorganic fertilizer; Cn0, control.

b QC, quality control; SD, standard deviation.

### Data availability.

The sequence data for the rhizospheric soil metagenomes were deposited in the NCBI Sequence Read Archive (SRA) under the accession numbers SRX7764933 (control), SRX7764932 (8 tons/ha composted-manure treatment), SRX7764931 (4 tons/ha composted-manure treatment), SRX7764930 (60 kg/ha inorganic fertilizer treatment), and SRX7764929 (120 kg/ha inorganic fertilizer treatment). The BioProject number is PRJNA607213.
